# Tightening Up the Control of Treadmill Walking: Effects of Maneuverability Range and Acoustic Pacing on Stride-to-Stride Fluctuations

**DOI:** 10.3389/fphys.2019.00257

**Published:** 2019-03-22

**Authors:** Melvyn Roerdink, Christa P. de Jonge, Lisette M. Smid, Andreas Daffertshofer

**Affiliations:** Department of Human Movement Sciences, Faculty of Behavioural and Movement Sciences, Amsterdam Movement Sciences and Institute for Brain and Behavior Amsterdam, Vrije Universiteit Amsterdam, Amsterdam, Netherlands

**Keywords:** motor control, variability, redundancy, detrended fluctuation analysis, goal-equivalent manifold

## Abstract

The correlational structure of stride-to-stride fluctuations differs between healthy and pathological gait. Uncorrelated and anti-persistent stride-to-stride fluctuations are believed to indicate pathology whereas persistence represents healthy functioning. However, this reading can be questioned because the correlational structure changes with task constraints, like acoustic pacing, signifying the tightness of control over particular gait parameters. We tested this “tightness-of-control interpretation” by varying the maneuverability range during treadmill walking (small, intermediate, and large walking areas), with and without acoustic pacing. Stride-speed fluctuations exhibited anti-persistence, suggesting that stride speeds were tightly controlled, with a stronger degree of anti-persistence for smaller walking areas. Constant-speed goal-equivalent-manifold decompositions revealed simultaneous control of stride times and stride lengths, especially for smaller walking areas to limit stride-speed fluctuations. With acoustic pacing, participants followed both constant-speed and constant-stride-time task goals. This was reflected by a strong degree of anti-persistence around the stride-time by stride-length point that uniquely satisfied both goals. Our results strongly support the notion that anti-persistence in stride-to-stride fluctuations reflect the tightness of control over the associated gait parameter, while not tightly regulated gait parameters exhibit statistical persistence. We extend the existing body of knowledge by showing quantitative changes in anti-persistence of already tightly regulated stride-speed fluctuations.

## Introduction

Assessing the structure of correlations in stride-to-stride fluctuations has gained in popularity over the years (Hausdorff et al., 1996, 1997, 2000; Herman et al., 2005; Pierrynowski et al., 2005; Terrier et al., 2005; Delignières and Torre, 2009; Dingwell and Cusumano, 2010, 2015; Dingwell et al., 2010, 2018; Nessler et al., 2011; Decker et al., 2012, 2013; Sejdić et al., 2012; Terrier and Dériaz, 2012, 2013; Kaipust et al., 2013; Uchitomi et al., 2013; Marmelat et al., 2014; Rhea et al., 2014a,b; Roerdink et al., 2015; Bohnsack-McLagan et al., 2016; Terrier, 2016; Choi et al., 2017; Kuznetsov and Rhea, 2017). This may have been instigated by possible differences between healthy and pathological gait (Hausdorff et al., 1997, 2000; Goldberger et al., 2002; Herman et al., 2005; Uchitomi et al., 2013). The correlational structure of stride-to-stride fluctuations is often quantified with the detrended fluctuation analysis (DFA; Peng et al., 1993) to assess the relation between the magnitude of stride-to-stride fluctuations *F* and the length of the time interval *n* over which these fluctuations are observed. If the relation between *F* and *n* obeys a power law, i.e., if *F*(*n*)∝*n*^α^, then the correlations are scale-free and the corresponding α-exponent will indicate particular dependencies: if deviations are statistically more likely to be followed by subsequent deviations in the same direction, then a time series exhibits statistical persistence; that case implies α > 0.5. During over-ground walking, stride times, stride lengths, and stride speeds in healthy subjects exhibit such statistical persistence (Terrier et al., 2005). In contrast, for fall-prone elderly and patients with neurodegenerative diseases this persistence appears lost (Hausdorff et al., 1997, 2000; Goldberger et al., 2002; Herman et al., 2005; Uchitomi et al., 2013) and deviations become either entirely uncorrelated (α ≈ 0.5) or anti-persistent (α < 0.5; deviations are statistically more likely to be followed by deviations in the opposite direction). These between-group differences arguably led to the reading that uncorrelated and anti-persistent stride-to-stride fluctuations indicate aging, disease and pathology, while persistent ones indicate healthy physiological functioning (Goldberger, 1996; Goldberger et al., 2002).

While this interpretation arguably finds support when looking at physiological functions other than walking like heart-rate dynamics, respiration or postural control, as of yet it is not without dispute (Dingwell and Cusumano, 2010). Part of the criticism originates from studies that reveal qualitative within-subject changes in the correlational structure of stride-to-stride fluctuations as a function of task variations. For example, stride-time time series often change from persistence to anti-persistence when paced by a metronome (Hausdorff et al., 1996; Terrier et al., 2005; Delignières and Torre, 2009; Nessler et al., 2011; Sejdić et al., 2012; Terrier and Dériaz, 2012, 2013; Marmelat et al., 2014; Roerdink et al., 2015; Bohnsack-McLagan et al., 2016; Terrier, 2016; Choi et al., 2017), for both treadmill and over-ground walking alike. A similar qualitative change from persistence to anti-persistence has been found in stride-length time series when foot placement is imposed with a sequence of stepping targets (Bohnsack-McLagan et al., 2016; Terrier, 2016). Stride-speed time series become anti-persistent when walking on a fixed-speed motorized treadmill (Dingwell and Cusumano, 2010, 2015; Dingwell et al., 2010, 2018; Terrier and Dériaz, 2012, 2013; Roerdink et al., 2015; Bohnsack-McLagan et al., 2016; Terrier, 2016) instead of on a self-paced motorized treadmill (Choi et al., 2017) or over ground (Terrier et al., 2005). The degree of anti-persistence in stride speeds during treadmill walking appears also to be affected by cognitive dual-tasking (Decker et al., 2013). Such task-variation differences—observed in healthy adults—led Dingwell and Cusumano (2010) to propose an alternative interpretation: anti-persistence in stride-to-stride fluctuations reflects the tightness of control over the associated gait parameter; deviations are rapidly corrected in subsequent strides. In turn, gait parameters that are not tightly regulated exhibit statistical persistence, that is, deviations are allowed to persist over multiple consecutive strides. Here, we would like to note that this “tightness-of-control interpretation” is not necessarily incompatible with the breakdown of statistical persistence in the various gait parameters observed with aging and disease, which may simply reflect the need to tightly regulate the associated gait parameter (“cautious control”; Dingwell and Cusumano, 2010).

In the current study on acoustically paced and unpaced treadmill walking, we further examined the tightness-of-control interpretation. In line with previous studies (Hausdorff et al., 1996; Terrier et al., 2005; Delignières and Torre, 2009; Nessler et al., 2011; Sejdić et al., 2012; Terrier and Dériaz, 2012, 2013; Marmelat et al., 2014; Roerdink et al., 2015; Bohnsack-McLagan et al., 2016; Terrier, 2016; Choi et al., 2017), we expected a qualitative change from persistence to anti-persistence in stride-time fluctuations with acoustic pacing. However, we also aimed at finding more subtle effects by experimentally varying the demands to tightly regulate stride-speed fluctuations. We did this by reducing the maneuverability range along the treadmill, thereby experimentally increasing the demands for tightly regulating walking speed, which was expected to result in a stronger degree of anti-persistence in stride-speed fluctuations (i.e., lower α-values). One way to regulate stride speed, and thereby preventing large positional displacements along the treadmill (Dingwell and Cusumano, 2010, 2015; Roerdink et al., 2015), is to couple stride-length and stride-time deviations (Dingwell and Cusumano, 2010, 2015; Terrier and Dériaz, 2012; Roerdink et al., 2015). Evidence for this coupling has recently been suggested by similar α-values for cross-correlated phase-randomized stride-speed surrogates and original stride-speed time series (Dingwell and Cusumano, 2010; Terrier and Dériaz, 2012; Roerdink et al., 2015). However, such a surrogate analysis cannot unravel the subtle changes in stride-length and stride-time regulation with task variations. This calls for a constant-speed goal-equivalent-manifold (GEM) decomposition (Dingwell and Cusumano, 2010, 2015; Decker et al., 2012; Cusumano and Dingwell, 2013; Bohnsack-McLagan et al., 2016; Dingwell et al., 2018), which specifies the many combinations of stride lengths and stride times resulting in a constant speed. In line with previous studies (Dingwell and Cusumano, 2010, 2015; Decker et al., 2012; Cusumano and Dingwell, 2013; Bohnsack-McLagan et al., 2016; Dingwell et al., 2018), we expected a smaller variation and anti-persistence in deviations perpendicular to the constant-speed GEM (i.e., representing combinations of stride lengths and stride times resulting in speed deviations that will be rapidly corrected) than along the GEM (i.e., representing combinations of stride lengths and stride times resulting in a constant speed that are allowed to persist). When experimentally reducing maneuverability ranges along the treadmill, we further expected a stronger degree of anti-persistence in the deviations perpendicular to the constant-speed GEM (i.e., lower α-values). With acoustic pacing, we similarly anticipated anti-persistence, but then in both tangential and perpendicular deviations to meet both task goals simultaneously (i.e., constant speed and constant stride time).

## Materials and Methods

### Participants

Twenty-four healthy volunteers (19 female, age 23 ± 2 years, weight 66 ± 9 kg, height 1.73 ± 0.09 m, mean ± standard deviation) participated in the experiment.

### Experimental Setup

Participants walked on a motorized treadmill in which a single, 0.70 m wide by 3.00 m long force platform was embedded (sampling rate 200 Hz; C-Mill 3N by Motekforce Link, Culemborg/Amsterdam, The Netherlands). The treadmill was equipped with a projector to present different walking areas onto the walking surface. Computer-generated acoustic pacing stimuli (pitch 1,000 Hz, duration 20 ms) were played through speakers.

### Experimental Procedure

Participants were familiarized with treadmill walking by playing a walking-adaptability game for 5 min implemented in the accompanying CueFors software. Next, the preferred walking speed of participants was determined using an established procedure (Dingwell and Marin, 2006). We defined preferred walking speed as the most pleasant speed at which participants would prefer to walk for at least an hour (4.3 ± 0.4 km/h). Subsequently, a pre-experimental trial of 60 s was performed at this preferred walking speed to determine the self-selected step length (68.8 ± 5.9 cm) and cadence (52.8 ± 3.5 strides/min) of participants.

In the repeated-measurements experiment proper, we varied the maneuverability range by projecting small (120% of the self-selected step length of participants), intermediate (215% of the self-selected step length), or large (3 m) walking areas (all with a width of 0.7 m) onto the walking surface. Participants were instructed to start walking in the center of the imposed walking area and to look in forward direction while walking. Walking area conditions were performed with and without isochronous acoustic pacing (beats per minute set in accordance to the self-selected cadence of participants at their preferred walking speed; in strides/min). For the pacing conditions, participants were instructed to accurately synchronize their left heel strikes with the metronome beeps. The resultant six experimental conditions were counterbalanced over the 24 participants. Specifically, half of the participants started with a block of acoustic pacing trials, the other half started with a block of trials without acoustic pacing. Within each block, the three walking area conditions (i.e., 1. small, 2. intermediate, 3. large) were counterbalanced, so yielding six possible orders (i.e., 123, 132, 213, 231, 312, 321). In combination, the fully counterbalanced design implied that all 12 possible orders of the six experimental conditions occurred twice within our sample of 24 participants. See Supplementary Material for a video showing the six experimental conditions. Each condition lasted until 280 strides were completed. Previous studies suggest that this is an adequate amount of strides to determine scaling exponents with excellent within-day test-retest reliability (Pierrynowski et al., 2005) and with sufficient statistical power (Kuznetsov and Rhea, 2017). Condition duration depended on the self-selected cadence of participants. Between conditions, participants rested for at least 2 min. The total experiment lasted ~ 70 min per participant.

### Data Analyses

Left stride-time, stride-length and stride-speed time series were derived from online determined left heel-strike events (Roerdink et al., 2008) and associated anterior-posterior center-of-pressure positions (Roerdink et al., 2014). Specifically, stride times (ST in s) were defined as the time intervals between consecutive heel-strike events. Stride lengths (SL in m) were derived by multiplying these stride times with the belt speed (in m/s), while correcting for spatial separation in consecutive ipsilateral heel-strike positions on the treadmill surface (Roerdink et al., 2014). Finally, we determined stride speeds (SS in m/s) as the ratio of stride length over stride time for each stride cycle (Dingwell and Cusumano, 2010; Roerdink et al., 2015; Terrier, 2016). Since participants generally require a certain number of strides to reach synchronization with the beat of a metronome (Roerdink et al., 2011), the first 20 stride cycles were excluded for both paced and unpaced conditions. The next 256 stride cycles were used for further analyses, yielding ST, SL and SS time series of 256 data points.

**Figure 1:**
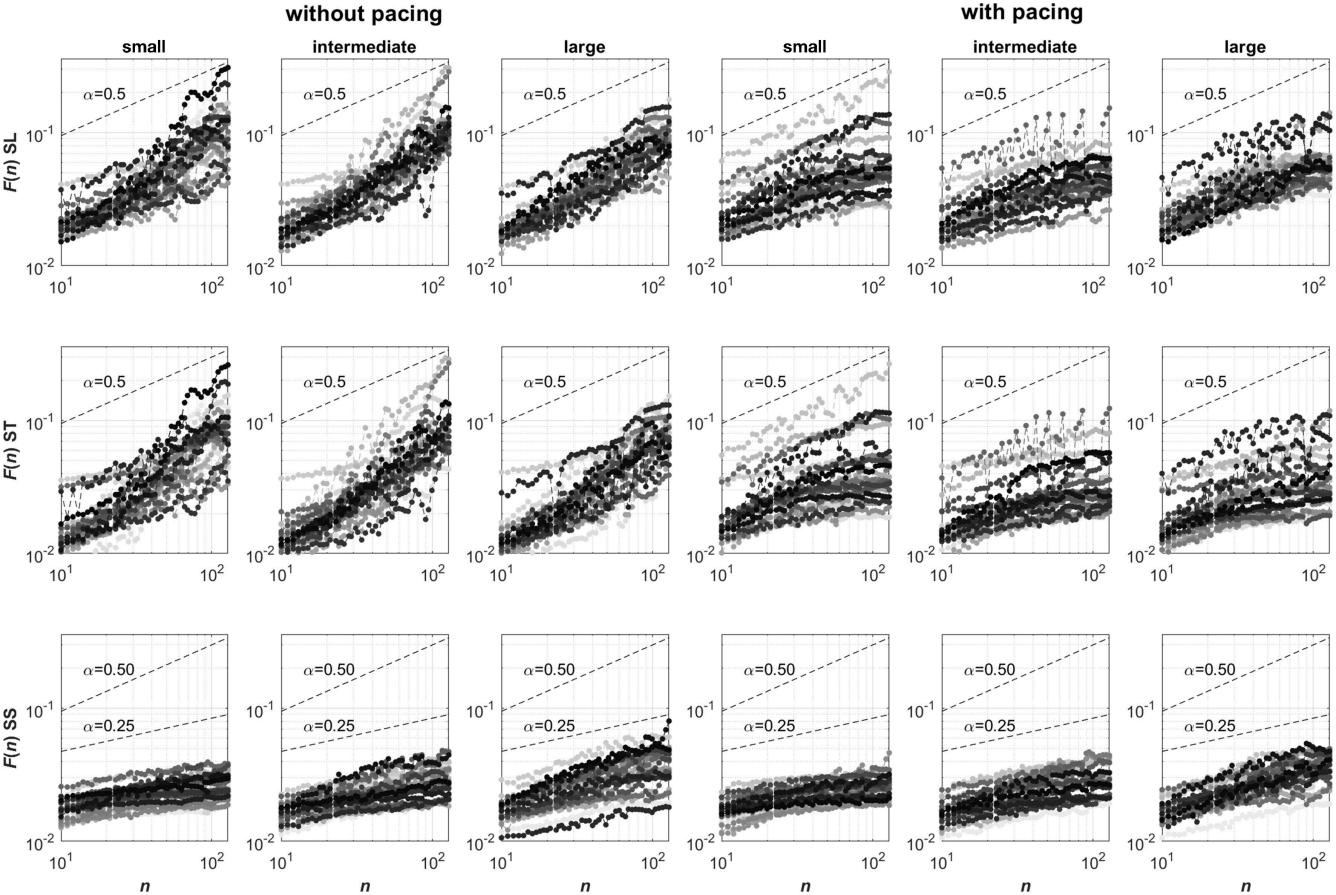
Log-log representations of *F* against *n* (ranging from *n* = 10 to *n* = 128) for stride-length (SL, top row), stride-time (ST, center row) and stride-speed (SS, bottom row) series. Columns represent the six Pacing by Walking Area conditions. In each panel, 24 curves are depicted with a unique grayscale for each participant. Note that the curves for stride speeds consistently have slopes smaller than 0.5, suggesting anti-persistence in the series, whose degree seemingly varied over the three walking area conditions. The curves for stride-length and stride-time series are less consistent but overall seemed to have slopes greater than 0.5 without pacing (suggesting persistence) and smaller than 0.5 with pacing (suggesting anti-persistence), without clear effects of the walking-area manipulation. The strong resemblance in the stride-length and stride-time representations of individual participants may point to a strong coupling between these two gait parameters.

For these time series, the DFA scaling exponents were determined (Peng et al., 1993). DFA is an established method with performance and limitations being systematically studied (see e.g., Hu et al., 2001; Chen et al., 2002, 2005; Ma et al., 2010; Xu et al., 2011). We first determined the mean square roots of linearly detrended residuals *F*(*n*) of the cumulative sum of the mean-centered time series for 50 exponentially spaced non-overlapping intervals of *n* data points. In Figure 1, log-log representations of *F* against *n* are depicted for all conditions and participants. The presence of power-law behavior in these representations was subsequently verified using a maximum likelihood approach testing for the appropriateness of a linear model when fitting log(*F*(*n*)) as a function of log(*n*) against a set of alternative models; see Ton and Daffertshofer (2016) for more detail. We considered power-law behavior to be present whenever the Bayesian information criterion was lowest for the linear model, which was the case for 204 of the 432 time series. This was deemed acceptable considering the relatively short time series, containing only 256 points each, which limits the reliability of the obtained probability densities of *F* for different intervals *n* (particularly so for larger *n*; Ton and Daffertshofer, 2016). Next, the DFA scaling exponent α (Peng et al., 1993) was determined by estimating the slope of the log-log representation of *F* against *n* (i.e., from *n* = 10 to *n* = 128 samples). Finally, the maximal displacement along the treadmill (MAD) was determined, defined as the maximal absolute value of the cumulative sum of mean-centered SS time series (Roerdink et al., 2015).

To explore the nature of the expected tighter control of SS with smaller walking areas, we performed surrogate analyses and a speed-GEM-based decomposition. With regard to the former, phase-randomization and cross-correlated phase-randomization surrogate techniques were applied to the SS time series, from which α was determined and compared to that of the original SS time series, similar to Dingwell and Cusumano (2010) and Roerdink et al. (2015). For all six conditions, we expected similar scaling exponents for original and cross-correlated phase-randomized SS time series but dissimilar scaling exponents for phase-randomized SS time series, which would reflect that deviations in ST and SL were adjusted conjointly instead of independently of one another (Dingwell and Cusumano, 2010; Terrier and Dériaz, 2012; Roerdink et al., 2015).

With regard to the decomposition of fluctuations relative to the constant-speed GEM defined as a diagonal line in the ST by SL plane, we followed the procedure outlined by Dingwell et al. (2010). In brief, ST and SL time series were first normalized to unit variance. Subsequently, a linear coordination transformation was performed to obtain deviations tangential (δ_T_) and perpendicular (δ_P_) to the constant-speed GEM. Finally, standard deviations σ and scaling exponents α were determined from the so-obtained δ_T_ and δ_P_ series. This normalization procedure provides a clear reference (σ = 1) for comparisons of the magnitude of the fluctuations along and perpendicular to the GEM: we expected σ(δ_T_) to be greater than 1 and σ(δ_P_) to be smaller than 1. As said, we expected α(δ_P_) to be smaller than 0.5, with a stronger degree of anti-persistence for smaller walking areas (i.e., lower α(δ_P_)-values). With acoustic pacing, anti-persistence in both α(δ_P_) and α(δ_T_) was expected, reflecting an attempt to constrain all variability to the intersection between the diagonal constant-speed GEM and the vertical constant-stride-time line to satisfy both goals simultaneously.

### Statistics

The scaling exponents α were subjected to one-sample *t*-tests against 0.5 (i.e., α-value reflecting uncorrelated noise) to confirm statistical persistence or anti-persistence in ST, SL, SS, δ_P_, and δ_T_ time series. The effects of Walking Area and Acoustic Pacing were examined with a 3 (small, intermediate, large walking areas) × 2 (with and without pacing) repeated-measures ANOVA, separately for all outcome measures. Whenever the sphericity assumption was violated, degrees of freedom were adjusted with Greenhouse-Geisser (for ε < 0.75) or Huynh-Feldt (for ε ≥ 0.75) methods. Effects were labeled significant if *p* < 0.05. Effect sizes were expressed as partial η^2^. *Post-hoc* analyses entailed two-tailed paired-samples *t*-tests.

## Results

### DFA Scaling Exponents α and Maximal Absolute Displacement (MAD)

**Figure 2:**
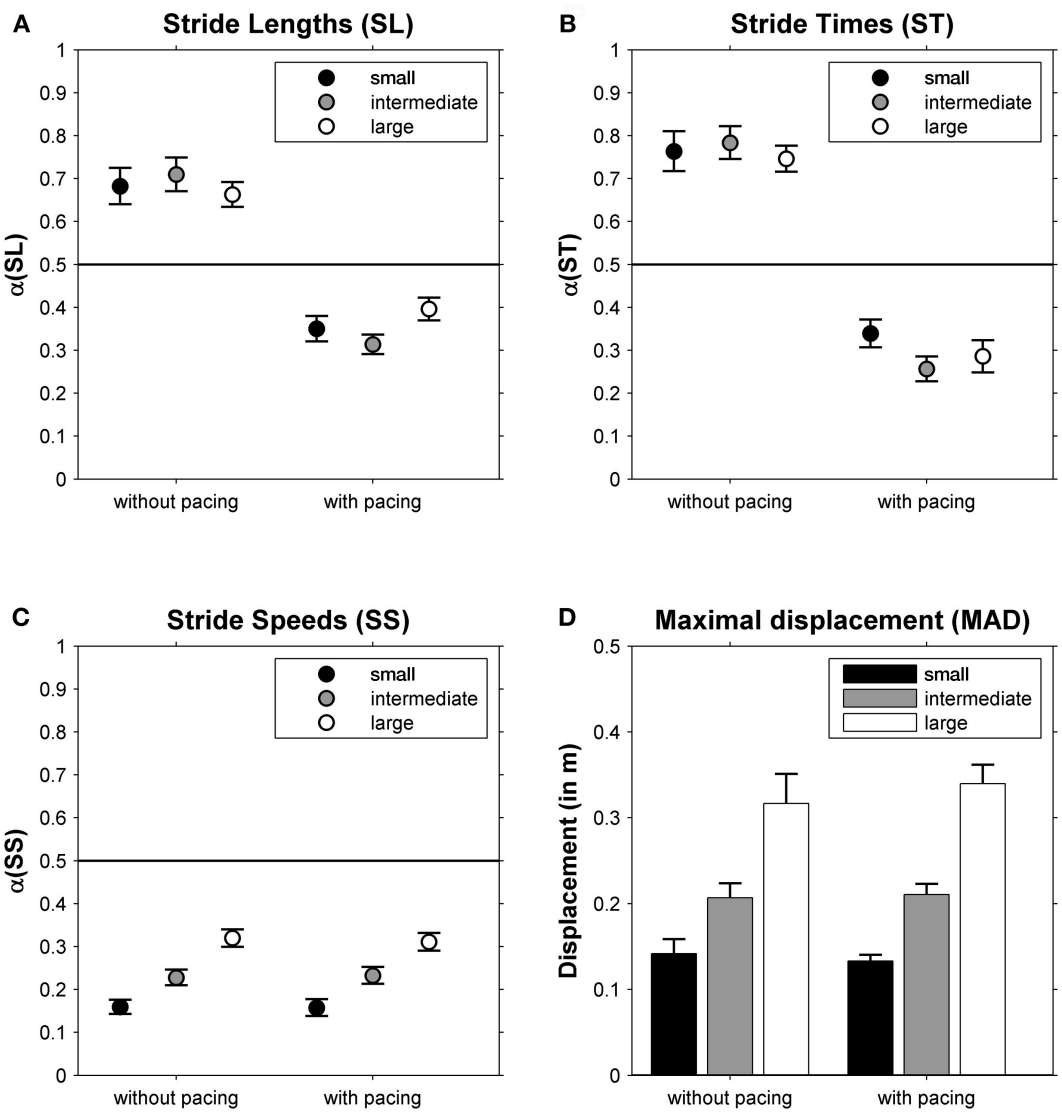
The correlational structure of stride-to-stride fluctuations in stride lengths (α(SL), **A**), stride times (α(ST), **B**) and stride speeds (α(SS), **C**) is depicted as a function of Pacing and Walking Area. In **(D)**, the maximal absolute displacement along the treadmill is depicted for these conditions. Error bars represent standard errors.

The DFA scaling exponents α of SL, ST and SS are depicted in Figures 2A–C, respectively. One-sample *t*-tests revealed that all α-values differed significantly from 0.5 (all |*t*_(23)_| > 3.92, all *p* < 0.001). For SL and ST a main effect of Pacing was observed (see for statistics Table 1): stride-to-stride fluctuations changed from persistence (without pacing) to anti-persistence (with pacing; Figures 2A,B). For SS a main effect of Walking Area was observed (Table 1). Stride-to-stride fluctuations for SS showed anti-persistence in all conditions, but the degree of anti-persistence varied over walking areas (Figure 2C), with significant *post-hoc* differences between all three walking areas (all *t*_(23)_ > 5.36, *p* < 0.001). For MAD, also a main effect of Walking Area was observed (Table 1), with smaller displacement along the treadmill for smaller walking areas (Figure 2D) as evidenced by significant *post-hoc* differences between all three walking areas (all *t*_(23)_ > 4.62, *p* < 0.001).

**Table 1:** Main and interaction effects of the repeated-measures ANOVA with the factors Walking Area and Pacing for all dependent variables.

	**Walking area**	**Pacing**	**Walking area × Pacing**
α(SL)	*F*_(2, 46)_ = 0.33, *p* = 0.718, η^2^ = 0.014	***F*_(1, 23)_ = 111.26, *p* < 0.001, *η*^2^ = 0.829**	*F*_(2, 46)_ = 2.95, *p* = 0.062, η^2^ = 0.114
α(ST)	*F*_(2, 46)_ = 1.49, *p* = 0.237, η^2^ = 0.061	***F*_(1, 23)_ = 150.95, *p* < 0.001, *η*^2^ = 0.868**	*F*_(2, 46)_ = 1.96, *p* = 0.152, η^2^ = 0.079
α(SS)	***F*_(2, 46)_ = 49.26, *p* < 0.001, *η*^2^ = 0.682**	*F*_(1, 23)_ = 0.02, *p* = 0.891, η^2^ = 0.001	*F*_(2, 46)_ = 0.19, *p* = 0.831, η^2^ = 0.008
MAD	***F*_(1.28, 29.43)_ = 47.81, *p* < 0.001, *η*^2^ = 0.675**	*F*_(1, 23)_ = 0.21, *p* = 0.652, η^2^ = 0.009	*F*_(1.61, 36.95)_ = 0.40, *p* = 0.629, η^2^ = 0.017
σ(δ_T_)	*F*_(2, 46)_ = 2.40, *p* = 0.102, η^2^ = 0.095	*F*_(1, 23)_ = 0.02, *p* = 0.895, η^2^ * =* 0.001	*F*_(1.42, 32.71)_ = 0.70 *p* = 0.458, η^2^ = 0.029
σ(δ_P_)	*F*_(2, 46)_ = 1.67, *p* = 0.199, η^2^ = 0.068	*F*_(1, 23)_ < 0.01, *p* = 0.989, η^2^ < 0.001	*F*_(1.36, 31.32)_ = 0.67 *p* = 0.466, η^2^ = 0.028
α(δ_T_)	*F*_(2, 46)_ = 0.39, *p* = 0.679, η^2^ = 0.017	***F*_(1, 23)_ = 157.06, *p* < 0.001, *η*^2^ = 0.872**	*F*_(2, 46)_ = 2.20, *p* = 0.122, η^2^ = 0.087
α(δ_P_)	***F*_(2, 46)_ = 52.90, *p* < 0.001, *η*^2^ = 0.697**	*F*_(1, 23)_ < 0.01, *p* = 0.992, η^2^ < 0.001	*F*_(2, 46)_ = 0.09, *p* = 0.918, η^2^ = 0.004

*Significant effects are presented in bold font*.

### Surrogate Results for Stride Speeds and Its GEM-Based Decomposition

**Figure 3:**
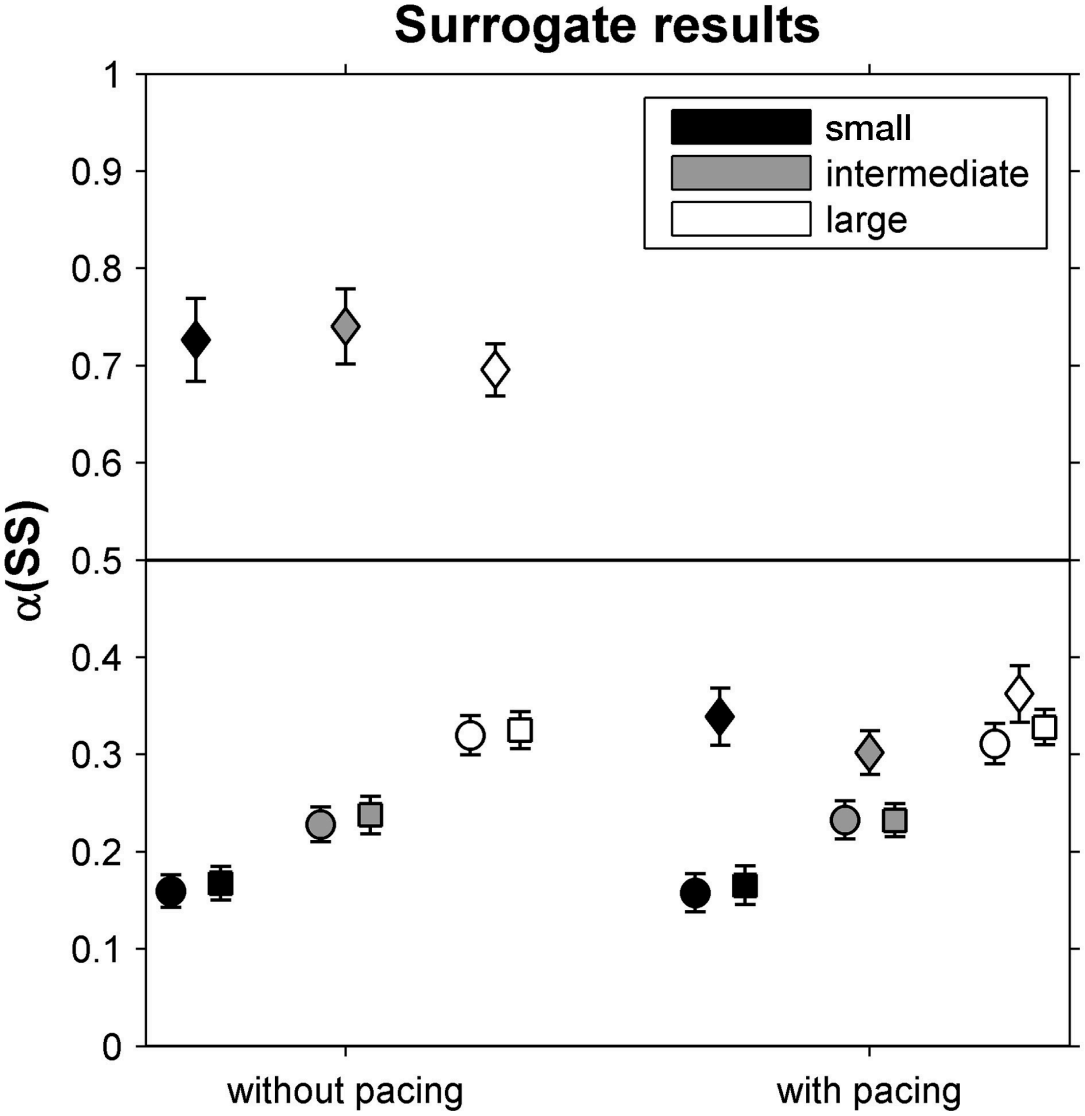
DFA scaling exponents α(SS) for original stride-speed time series (circles) as well as for phase-randomized surrogates (diamonds) and cross-correlated phase-randomized surrogates (squares), depicted as a function of Pacing and Walking Area conditions. Error bars represent standard errors.

In Figure 3, α-values are depicted for original and surrogate SS time series. While α-values differed markedly between phase-randomized and original time series for all but the large-walking-area-with-pacing condition, they were similar for cross-correlated phase-randomized surrogates and original SS time series for all six conditions, suggesting that participants coupled ST and SL deviations to limit variations in SS.

**Figure 4:**
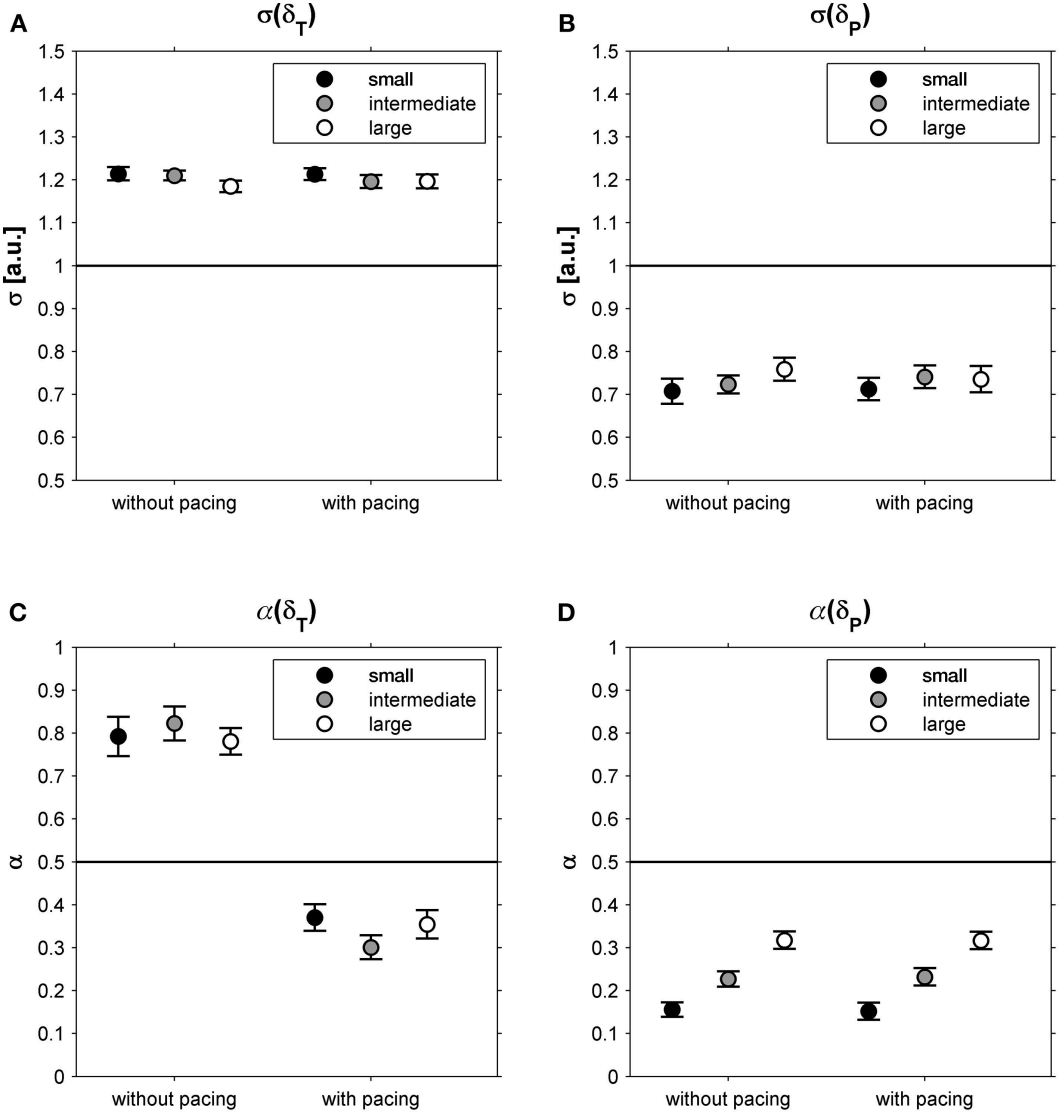
Relative standard deviations (σ) for deviations tangential (δ_T_; **A**) and perpendicular (δ_P_; **B**) to the constant-speed GEM, depicted as a function of Pacing and Walking Area. In **(C,D)**, the corresponding DFA scaling exponents α are shown for these conditions. Error bars represent standard errors.

This suggestion was supported by the observation that participants exhibited far greater variability along the constant-speed GEM (σ(δ_T_) = 1.20; 95% CI: 1.18-1.23; Figure 4A) than perpendicular to it (σ(δ_P_) = 0.73; 95% CI: 0.69-0.77; Figure 4B), for all conditions alike (Table 1). One-sample *t*-tests revealed that for all conditions α(δ_T_) and α(δ_P_) differed significantly from 0.5 (all |*t*_(23)_| > 4.15, all *p* < 0.001). Interestingly, the correlational structure of δ_P_ time series varied significantly with walking area, whereas for δ_T_ no such effect was observed (Table 1): stride-to-stride fluctuations for δ_P_ showed anti-persistence across conditions, with stronger corrections (i.e., lower α(δ_P_)-values) for smaller walking areas (Figure 4D), as evidenced by significant *post-hoc* differences between all three walking areas (all *t*(_23)_ > 4.84, *p* < 0.001). Apparently, participants tried to regulate stride speed by correcting δ_P_ deviations from each stride to the next, and more tightly so for smaller walking areas. The correlational structure of δ_T_ time series varied significantly with acoustic pacing (Table 1): without pacing participants allowed δ_T_ deviations to persist across multiple strides (i.e., α(δ_T_) > 0.5), whereas with pacing δ_T_ deviations became anti-persistent (α(δ_T_) < 0.5; Figure 4C).

## Discussion

We examined the effects of maneuverability range and acoustic pacing on the correlational structure of stride-to-stride fluctuations during treadmill walking in a group of healthy, mostly female, young adults. For all six conditions we found anti-persistence in stride-speed fluctuations. This suggests that this gait parameter is under tight control (Dingwell and Cusumano, 2010, 2015; Dingwell et al., 2010, 2018; Terrier and Dériaz, 2012; Decker et al., 2013; Roerdink et al., 2015; Bohnsack-McLagan et al., 2016; Terrier, 2016). Interestingly, the degree of anti-persistence varied significantly with manipulations in the maneuverability range: a stronger degree of anti-persistence was observed for smaller walking areas (Figure 2C). Apparently, stride-speed deviations were always rapidly corrected during treadmill walking, yet most compellingly so for smaller walking areas. The success of this tighter control of stride-speed fluctuations was corroborated by smaller absolute displacements along the treadmill with smaller walking areas (Figure 2D). We also found a qualitative change in the correlational structure of stride-time fluctuations, from persistence without pacing to anti-persistence with pacing, an expected finding in line with previous observations (Terrier et al., 2005; Delignières and Torre, 2009; Sejdić et al., 2012; Terrier and Dériaz, 2012, 2013; Marmelat et al., 2014; Roerdink et al., 2015; Terrier, 2016).

To further assess the regulation of stride-to-stride fluctuations, we complemented the analysis by comparing α between original stride-speed time series and surrogates (Dingwell and Cusumano, 2010; Terrier and Dériaz, 2012; Roerdink et al., 2015) and by an examination of a constant-speed GEM decomposition (Dingwell and Cusumano, 2010, 2015; Decker et al., 2012; Cusumano and Dingwell, 2013; Bohnsack-McLagan et al., 2016; Dingwell et al., 2018). With regard to the former, in all conditions we found α-values of only cross-correlated phase-randomized surrogates (and not those of the phase-randomized surrogates) to be similar to α-values of the original stride-speed time series (Figure 3). This suggests that participants simultaneously controlled both stride times and stride lengths in such a way that deviations in either variable were canceled out by concomitant changes in the other (Dingwell and Cusumano, 2010; Terrier and Dériaz, 2012; Roerdink et al., 2015). Such coupling between stride times and stride lengths limits stride-speed fluctuations and thereby displacement along the treadmill.

The constant-speed GEM decomposition results supported this interpretation. The magnitude of the fluctuations tangential to the GEM (i.e., representing combinations of stride lengths and stride times resulting in a constant speed) was much greater than that perpendicular to it (i.e., representing combinations of stride lengths and stride speeds resulting in speed variations; Figures 4A,B), a finding consistent with previous studies (Dingwell and Cusumano, 2010, 2015; Decker et al., 2012; Cusumano and Dingwell, 2013; Bohnsack-McLagan et al., 2016; Dingwell et al., 2018). A new insight that the constant-speed GEM decomposition revealed was that adherence to the constant-speed goal was scalable, becoming more manifest with smaller walking areas, as evidenced by a stronger degree of anti-persistence in deviations perpendicular to the GEM (Figure 4D). Thus, not only were fluctuations perpendicular to the constant-speed GEM smaller in magnitude, these fluctuations were also rapidly corrected (or reversed) to limit speed variations, most compellingly so for the smaller walking areas.

With acoustic pacing, an explicit task goal had been added to the constant-speed goal: a constant stride time had to be achieved as prescribed by the metronome. This yielded a qualitative change in the correlational structure of the fluctuations tangential to the constant-speed GEM (Figure 4C): from persistence without pacing to anti-persistence with pacing. It appears that with acoustic pacing, participants attempted to constrain all variability to the intersection between the diagonal constant-speed line and the constant-stride-time line in the stride-length by stride-time plane, thereby simultaneously satisfying both task goals. Contrasting our current findings and interpretation, however, Bohnsack-McLagan et al. (2016) recently concluded that in such situations participants adopt an ‘intermediate' strategy to balance errors with respect to each goal function, without fully satisfying either. Note that, in contrast to our results, Bohnsack-McLagan et al. (2016) did not observe clear anti-persistence in stride-time fluctuations (α(ST)), which may be caused by a mixture of synchronizers and non-synchronizers in the group of participants, nor did they find anti-persistence in deviations tangential to the constant-speed GEM (α(δ_T_)), presumably for the same reason. Future studies should address these inconsistencies by, e.g., examining the effects of acoustic (and/or visual) pacing on the correlational structure of stride-to-stride fluctuations. Future studies may examine nonlinear properties of stride-to-stride fluctuations by decomposing them into magnitude and sign series (Ivanov et al., 2009) and/or explore correlations at even shorter time scales (Delignières and Marmelat, 2013) than examined here, utilizing, for example, the current dataset (see Supplementary Material). Moreover, locomotor modeling studies (e.g., Ashkenazy et al., 2002) could benefit from a comprehensive approach by adding stride length and stride speed series, and their goal-dependent relationships, to the typically used stride interval series. Ultimately, this will provide a comprehensive understanding of how participants control various combinations of multiple goal functions during walking (constant speed, constant stride length, constant stride time).

We can conclude that our results strongly support the interpretation proposed by Dingwell and Cusumano (2010; see also Terrier and Dériaz, 2012; Decker et al., 2013; Roerdink et al., 2015) that anti-persistence in stride-to-stride fluctuations does not necessarily indicate aging, disease and pathology, but rather reflects the tightness of control over the associated gait parameter (i.e., deviations are rapidly corrected in subsequent strides), while variables that are not tightly regulated show statistical persistence (i.e., deviations are allowed to persist). We extend the existing body of knowledge by not only showing qualitative changes from persistence to anti-persistence with walking-task variations (without and with pacing, respectively), but also by showing significant quantitative changes in the degree of anti-persistence of already tightly regulated stride-speed fluctuations (with changes in walking area). Surrogate analyses and constant-speed GEM decomposition revealed that stride times and stride lengths were simultaneously controlled in order to limit speed fluctuations, most compellingly so for smaller walking areas. With acoustic pacing, participants seemed to satisfy both constant-speed and constant-stride-time task goals, as evidenced by the observed strong degree of anti-persistence around the single combination of stride time and stride length that uniquely satisfied both task goals.

## Data Availability

All datasets, acquired and processed, for this study are included in the Supplementary Files.

## Ethics Statement

The study met all applicable standards for the ethics of experimentation with human participants and was approved by the ethics committee of the department of Human Movement Sciences, Vrije Universiteit Amsterdam. All participants provided written informed consent prior to the experiment, in accordance with the Declaration of Helsinki.

## Author Contributions

MR, CdJ, and LS contributed conception of the study. All authors contributed to the design of the study, manuscript revision, read and approved the submitted version. CdJ and LS performed the experiment. MR performed the analysis and wrote the manuscript.

### Conflict of Interest Statement

The authors declare that the research was conducted in the absence of any commercial or financial relationships that could be construed as a potential conflict of interest.

## Supplementary Material

Video S1 Video showing the six experimental conditions.

Data Sheet 1 Zip file containing acquired data (i.e., stride-speed, stride-time and stride-length series) and processed data (i.e., the eight outcome measures listed in Table 1) for 24 participants in six conditions.

## Abbreviations

DFA: detrended fluctuation analysis
ST: stride times
SS: stride speeds
SL: stride lengths
GEM: goal-equivalent manifold
MAD: maximal displacement along the treadmill
*F*(*n*): magnitude of stride-to-stride fluctuations *F* over a given interval *n*
α: scaling exponent indicating correlational dependencies in time series
σ: standard deviation
δ_T_: deviations tangential to the constant-speed GEM
δ_P_: deviations perpendicular to the constant-speed GEM.
