# Supplementary material for: Tightening Up the Control of Treadmill Walking: Effects of Maneuverability Range and Acoustic Pacing on Stride-to-Stride Fluctuations
Source: Front Physiol. 2019 Mar 22;10:257. doi: 10.3389/fphys.2019.00257 (PMC6440225; doi:10.3389/fphys.2019.00257)
Supplement: Data Sheet 1 — Zip file containing acquired data (i.e., stride-speed, stride-time and stride-length series) and processed data (i.e., the eight outcome measures listed in Table 1) for 24 participants in six conditions. [file Data_Sheet_1.ZIP › SupplementaryFile_data/Readme.pdf]

This folder contains two Matlab data files corresponding to the manuscript by Melvyn Roerdink, Christa P. de Jonge, Lisette M. Smid & Andreas Daffertshofer submitted to the section Fractal Physiology of Frontiers in Physiology on the effects of maneuverability range and acoustic pacing on stride-to-stride dynamics in treadmill walking.

1) *rawdataRoerdinketal_FrontiersPhysiol_2018.dat*

   Comprises 3 struct files (DATST, DATSL and DATSS) containing time series of original (O, 256x1) stride times (ST), stride lengths (SL) and stride speeds (SS) series. DATST is a 1x24 struct array (1 for each of the 24 participants) with field C for each of the 6 conditions (1: pacing short, 2: pacing intermediate, 3 pacing large, 4: no pacing short, 5 no pacing, intermediate, 6 no pacing large) with field O (original series, 256 x 1) for the raw stride time time series. Same for DATSL and DATSS.

2) *statsdataRoerdinketal_FrontiersPhysiol_2018*

   Comprises 24x6 matrices for the 8 outcome measures listed in Table 1, from top to bottom: alphaSL, alphaST, alphaSS, MAD, STDT, STDP, alphaT, and alphaP. Rows represent the 24 participants, columns the 6 conditions in the following order: 1: pacing short, 2: pacing intermediate, 3 pacing large, 4: no pacing short, 5 no pacing, intermediate, 6 no pacing large.
